# Superconductivity–Electron Count Relationship in Heusler Phases—the Case of LiPd_2_Si

**DOI:** 10.1021/acs.chemmater.3c02398

**Published:** 2024-02-15

**Authors:** Karolina Górnicka, Xin Gui, Juan R. Chamorro, Tyrel M. McQueen, Robert J. Cava, Tomasz Klimczuk, Michał J. Winiarski

**Affiliations:** † Faculty of Applied Physics and Mathematics and Advanced Materials Centre, Gdansk University of Technology, ul. Narutowicza 11/12, 80-233 Gdańsk, Poland; ‡ Department of Chemistry, Princeton University, Princeton, New Jersey 08540, United States; § Materials Department and Materials Research Laboratory, University of California, Santa Barbara, Santa Barbara, California 93106, United States; ∥ Department of Chemistry, William H. Miller III Department of Physics and Astronomy, Department of Materials Science and Engineering, and Institute for Quantum Matter, Johns Hopkins University, Baltimore, Maryland 21218, United States

## Abstract

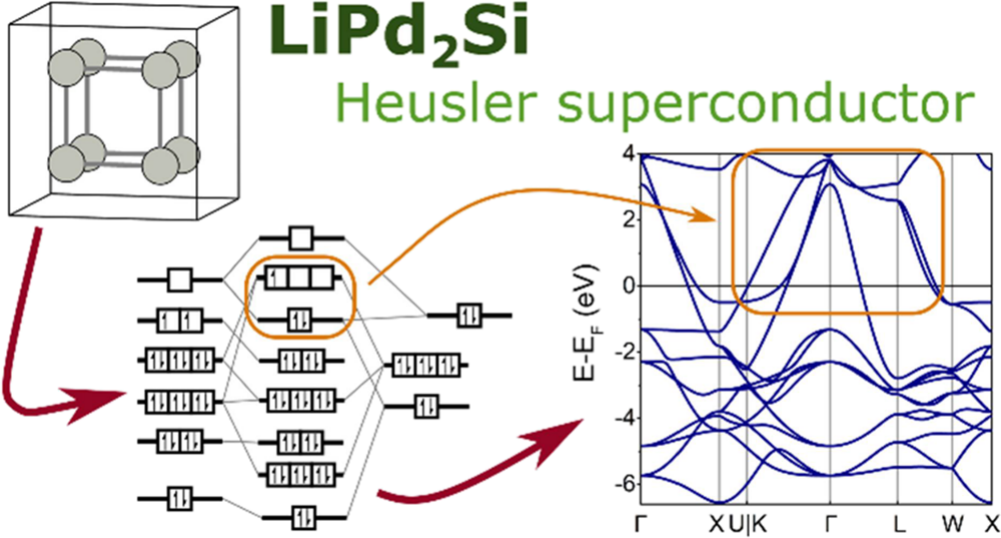

We report superconductivity in the full Heusler compound LiPd_2_Si (space group *Fm3̅m*, No. 225) at a critical temperature of *T*_c_ = 1.3 K and a normalized heat capacity jump at *T*_c_, Δ*C*/γ*T*_c_ = 1.1. The low-temperature isothermal magnetization curves imply type-I superconductivity, as previously observed in LiPd_2_Ge. We show, based on density functional theory calculations and using the molecular orbital theory approach, that while LiPd_2_Si and LiPd_2_Ge share the Pd cubic cage motif that is found in most of the reported Heusler superconductors, they show distinctive features in the electronic structure. This is due to the fact that Li occupies the site which, in other compounds, is filled with an early transition metal or a rare-earth metal. Thus, while a simple valence electron count–property relationship is useful in predicting and tuning Heusler materials, inclusion of the symmetry of interacting frontier orbitals is also necessary for the best understanding.

## Introduction

At the beginning of the last century, Fritz Heusler studied various manganese alloys and found Mn_2_CuAl—the first room-temperature ferromagnetic intermetallic compound that did not contain ferromagnetic elements.^1^ While the discovery of a ferromagnet not containing any ferromagnetic element was considered remarkable by itself, it turned out to be just the tip of an iceberg. The Mn_2_CuAl (“Full” Heusler) type is one of the most ubiquitous intermetallic structures^2−4^ and has been dubbed as the *metallic equivalent of the perovskite*.^5^ In addition, a number of closely related crystal structure types exist: the half Heusler (MgAgAs-type), inverse Heusler (CuHg_2_Ti-type),^4^ double half Heusler,^6^ and LiMgPdSb-type^7,8^ with a variety of chemical compositions and physical behaviors. The (full) Heusler intermetallic family is the fourth most common structure among more than 1000 structure types and more than 13,000 ternary intermetallic compounds.^2,5,9^ A wide range of properties is observed in that vast group of compounds including half metallic ferromagnetism,^3,10,11^ heavy Fermion behavior,^12−16^ shape memory effect and mechanocaloric coupling,^17,18^ and superconductivity.^19−23^

Full Heusler compounds form in the face-centered cubic crystal structure (space group *Fm3̅m*, no. 225) and can be viewed as a NaCl-type lattice (strukturbericht B1), with the extra atoms filling all of its tetrahedral voids. In Mn_2_CuAl, the sublattice is formed by Cu and Al atoms, and the voids are filled with Mn. Alternatively, the Heusler structure can also be seen as an ordered ternary variant of the CsCl-type cell (strukturbericht B2; see e.g., refs (4 and 24) or as a CaF_2_-type ([anti]fluorite, strukturbericht C1).

**Figure 1:**
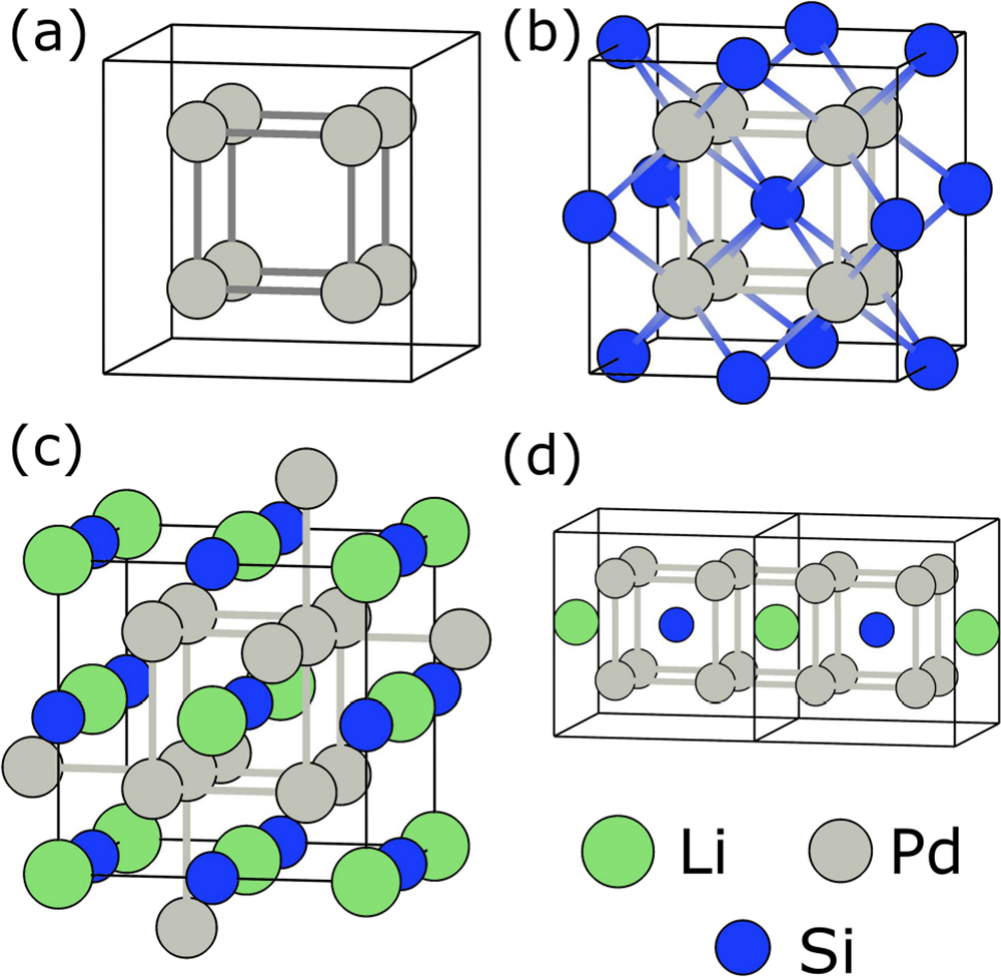
Crystal structure of a generic XY_2_Z full Heusler phase can be described by considering first a (Y_2_)_4_ cubic cage (a) which interacts with a FCC sublattice Z (panel b), forming a CaF_2_-type (strukturbericht C1) lattice. Full Heusler phase results from filling the voids of the CaF_2_-type lattice with atom X (panel c). The resulting structure can also be viewed as two interpenetrating CaF_2_-type networks (d). In LiPd_2_Si, the voids of the CaF_2_-type Pd_2_Si sublattice are filled with Li atoms that play the role of electron donors. As we discuss further, the electronic structure of the full Heusler phase can be traced back to the molecular orbitals of the cubic cage formed by Y atoms.

A general formula for the full Heusler compounds is XY_2_Z, where X and Y are usually transition metals and Z is a main-group element. In some compounds, X can be a rare earth element, alkaline (Li), or alkaline earth (Mg) metal. Within the unit cell, the eight Y atoms form a cubic cage, with each X atom neighboring six others (Figure 1a). Together the Z and Y_2_ sublattices form a CaF_2_-type Y_2_Z structure, with voids filled by X atoms (or conversely a XY_2_ structure with Z atoms filling the voids, as the X and Z sites (4*c* and 4*d*) are inequivalent but have the same symmetry.

Much of the diverse physical properties of the Heusler family can be rationalized within the framework of rigid band approximation and valence electron count (VEC). Within this simple approach, the electronic structure is assumed to be qualitatively robust toward changes in the elemental composition, and the difference between the properties thus stems from the change in the occupancy of different electronic states (i.e., the position of the Fermi level). While such a view is an obvious oversimplification, it is successful in explaining, for example, the effect of chemical doping on magnetic properties of Heusler compounds^4,5^ and the stability of XYZ-XY_2_Z half Heusler–full Heusler solid solutions.^25^ The use of molecular orbital (MO) theory, or more precisely crystal orbital (CO) theory, allows one to put the relationship between VEC and the physical properties into the context of chemical bonding. An example of this approach is presented in the recent literature,^4^ where it is explained that the exceptional stability and semiconducting properties of VEC = 8 and VEC = 18 half Heusler compounds by the closed shell configuration found for that electron count.^4^ They also provide, on the same grounds, an explanation of the semiconducting properties of several VEC = 24 full Heusler compounds,^4^ most notably VFe_2_Al.^26−29^

**Figure 2:**
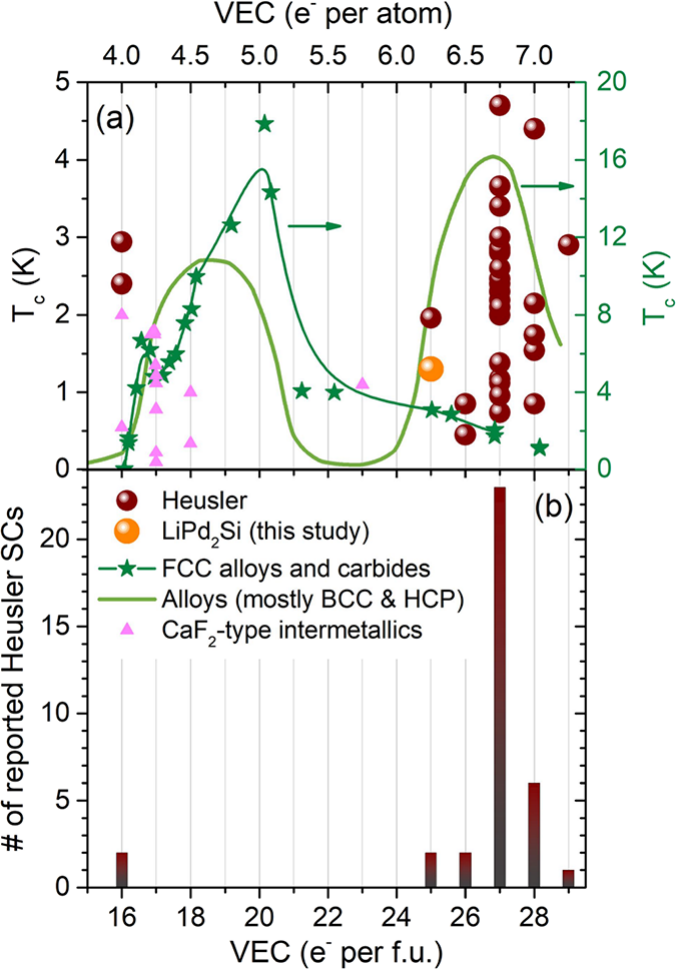
Panel (a) shows the highest reported *T*_c_ of Heusler superconductors for each VEC (brown spheres; LiPd_2_Si is shown in orange) compared to the reported *T*_c_–VEC relationship in crystalline BCC alloys^33^ (light green; data taken from ref (36)), FCC alloys and carbides (dark green stars; line is added as a guide for eyes), and CaF_2_-type intermetallics (pink triangles).^38^ Panel (b) shows the number of reported Heusler superconductors vs valence electron count.^24,30,31^

We have previously shown that all but two of the 34 known Heusler superconductors have VEC = 25–29, and 70% (24 of 34) have VEC = 27. The highest reported *T*_c_ of a Heusler SC is also found at that electron count^19,24,30,31^ (see Figure 2). In almost all of them, the X atom is a late *d*-block metal, i.e., Ni, Pd, Pt, and Au. Interestingly, VEC = 27 corresponds to 6.75 electrons per atom close to the second maximum of Matthias’ empirical *T*_c_–VEC relationship rule for pure elements and their alloys^32−34^ (see Figure 2a) and to the maximum of *T*_c_ for α-Mn-type high-entropy alloys.^35,36^ In our recent study, we reported superconductivity in MgPd_2_Sb, the occurrence of which was predicted based on the compound’s “magical” VEC = 27 electron count.^24^ Recent machine learning predictions of superconductivity have also identified several Heusler compounds with similar electron counts as possible superconductors.^37^

Recently, we also studied a series of compounds LiPd_2_Z (Z = Si, Ge, Sn; VEC = 25) and found superconductivity in LiPd_2_Ge with *T*_c_ = 1.96 K.^39^ The experimental results indicate that LiPd_2_Ge is a type-I weakly coupled BCS superconductor. Ab initio electron–phonon coupling calculations show a strong softening of the acoustic phonon mode in LiPd_2_*Z,* and since the effect is most pronounced in LiPd_2_Ge, we concluded that there is a correlation between superconductivity and a mode-softening effect. Our calculations predict superconductivity in two other members, LiPd_2_Si and LiPd_2_Sn, with the expected *T*_c_ below 1 K in both cases.^39^

In this paper, we report low-temperature studies of the superconducting properties of LiPd_2_Si—the second Heusler-type superconductor with VEC = 25. We also discuss its electronic structure and VEC in comparison to those of the remaining Heusler superconductors, highlighting the similarities and also pointing to the importance of changes in chemical bonding that violate the rigid band approximation.

## Experimental Details

**Figure 3:**
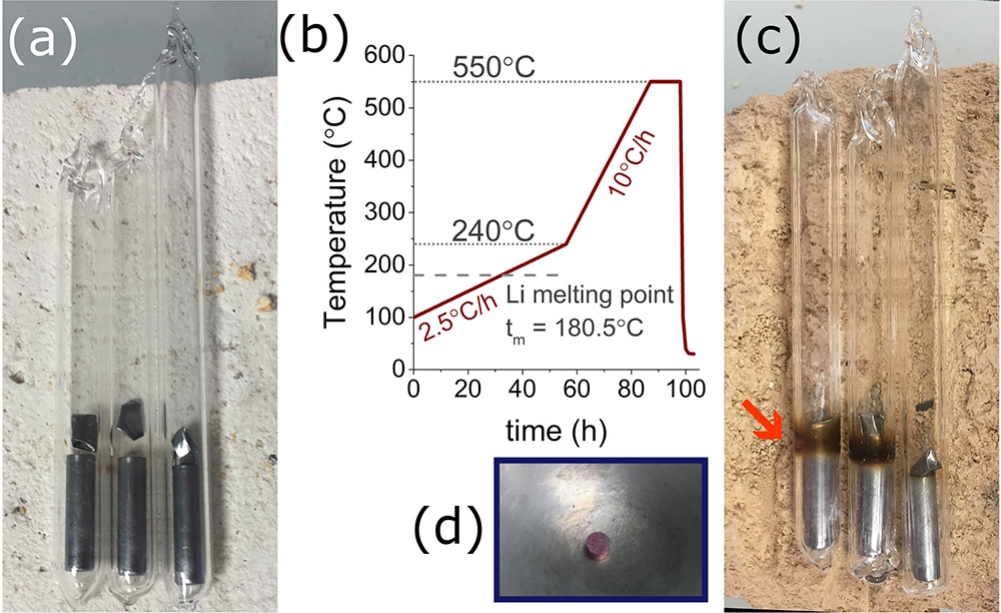
Panel (a) shows the ampules containing Ta crucibles loaded with pressed reagents (Li, Pd, and Si/Ge/Sn), capped with a piece of Ta foil. The heating procedure is shown in panel (b). Ampules after heating are shown in panel (c). Only a small discoloration due to the deposition of evaporated Li is seen on the tube walls. Panel (d) shows a resulting pellet of LiPd_2_Si with a brownish color. Results of measurements on the LiPd_2_Ge sample were recently presented in ref (39).

Polycrystalline samples of LiPd_2_Si and LiPd_2_Sn were synthesized by the two-step solid-state reaction method that we reported previously.^39^ Lithium chunks (Alfa Aesar, 99.99% pure), Pd powder (Mennica-Metale, Poland, 99.998%), Si pieces (Alfa Aesar, 99.9%), and Sn pellets (Alfa Aesar, 99.99%) were used as delivered without purification. Stoichiometric amounts of elemental reagents (with a 10% excess of Li to account for evaporative losses) were mixed and pressed into pellets using a hydraulic press inside a high-purity Ar-filled (p(O_2_) < 0.5 ppm) glovebox. The resulting pellets were placed in tantalum crucibles, covered with a piece of tantalum foil, and subsequently put in fused silica tubes that were evacuated, purged with argon gas, and backfilled with high-purity Ar prior to sealing (see Figure 3a). The use of a crucible cover and backfilling the ampules with Ar are necessary to avoid excessive Li evaporation during annealing (Figure 3c shows ampules after heating). The sealed tubes were then slowly heated at a rate of 2.5 °C/h from 100 to 240 °C (across the melting point of Li, *t*_m_ = 180.5 °C), then ramped at 10 °C/h to 550 °C, and soaked for 12 h (Figure 3b). Tubes were then air-quenched to room temperature and opened inside of the glovebox. Pellets were ground, repressed, and resealed in tubes, using the same Ta crucibles. The samples were then homogenized by annealing them at 610 °C over 3 days. The resulting dense, hard pellets were brownish in color (as previously reported for LiPd_2_Sn;^40,41^ see Figure 3d.

Heat capacity measurements employing the standard semiadiabatic pulse technique were conducted in the temperature range of 0.4–10 K using the ^3^He–^4^He option of the quantum design Dynacool physical property measurement system (PPMS). Magnetization measurements were performed with a QD MPMS-XL SQUID magnetometer in the temperature range of *T* = 0.4–1.8 K and in a magnetic field of up to μ_0_*H* = 0.5 T. Magnetization measurements performed on LiPd_2_Sn did not show any sign of superconducting transition down to *T* = 0.4 K, in agreement with our recent ab initio calculations.^39^

Resistivity measurement was performed on a polished fragment of a sample by means of the four-probe method, employing the ^3^He option of the PPMS to achieve *T* < 2 K temperatures. Thin platinum wires were mounted on the sample surface using a conductive silver paste.

Density functional theory (DFT) calculations of the electronic structure of LiPd_2_Si were done using the Quantum Espresso 6.7MaX code,^42−44^ employing the Perdew–Burke–Ernzerhof generalized gradient approximation (PBE GGA)^45^ of the exchange–correlation potential in the scalar relativistic approximation (neglecting spin–orbit coupling). Projector augmented-wave^46,47^ sets were taken from the PSlib^48^ library. The charge density and kinetic energy cutoff values were set to 500 and 55 Ry, respectively. The unit cell dimension was relaxed using the BFGS method yielding a = 5.9542 Å, reasonably close to the experimental value (*a* = 5.9059 Å^39^). Crystal orbital Hamilton population^49,50^ analysis was conducted using the Lobster 4.1.0 code^51,52^ using the basis set by Koga et al.^53^ for projections. Atomic charge analysis was performed using the BADER code.^54−56^ For comparison, band structures of four Heusler phases (YPd_2_In, LuPt_2_In, ZrNi_2_Ga, and HfPd_2_Al) were taken from the Materials Project database^57^ using the pymatgen module^58^ and are shown in the Supporting Information (Figure S4).

## Results and Discussion

The existence of the title compound, the physical properties of which are described in this paper, was recently reported by us in ref (39). The powder XRD pattern of LiPd_2_Si, together with the Rietveld structural analysis, is shown in Figure S1 of the Supporting Information. The profile refinement confirms a cubic *L2*_*1*_ Heusler-type crystal structure (space group *Fm3̅m*, No. 225) with the lattice parameter *a* = 5.9059(4) Å. Among the LiPd_2_Z series (Z = Si, Ge, Sn), LiPd_2_Si has the smallest unit cell, in agreement with the atomic radius trend of the tetrel elements.

**Figure 4:**
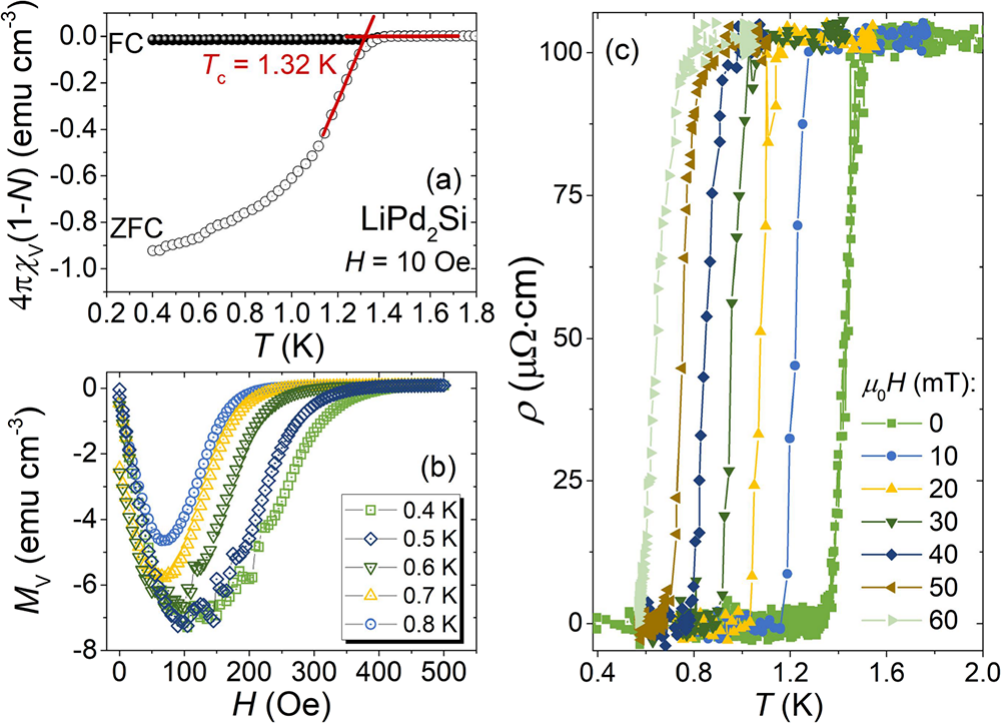
Panel (a) presents the temperature dependence of the volume magnetic susceptibility below 1.8 K, measured at 10 Oe with both ZFC and FC modes. Panel (b) shows field-dependent magnetization *M*_V_(*H*) measured below 500 Oe at various temperatures. Low-temperature resistivity measured at magnetic fields μ_0_*H* = 0 to 60 mT is shown in panel (c), showing an abrupt drop to zero at the critical temperature. See Figure S2 of the Supporting Information for resistivity data in a wider temperature range.

To characterize the low-temperature behavior of LiPd_2_Si, magnetic measurements were performed in different applied fields. Figure 4a displays the temperature dependence of the volume magnetic susceptibility χ_V_(T), defined as χ_V_ = *M*/*H* (*M—*magnetization, *H*—**applied magnetic field), upon both zero-field-cooled (ZFC) and field-cooled (FC) measurement modes in an applied low field of *H* = 10 Oe. The data are corrected for demagnetization effects and multiplied by 4π (here, a demagnetization factor of *N* = 0.58 is estimated from the *M*_V_(*H*) fit discussed later). An abrupt decrease of the magnetic susceptibility indicates the presence of a superconducting transition with *T*_c_ = 1.3 K, estimated as the point at which the extrapolation of the normal-state magnetic susceptibility intersects with the line set by the steepest slope of magnetization in the ZFC data set.^59^ The critical temperature obtained for LiPd_2_Si is larger than that predicted by ab initio calculations (*T*_c_ = 0.76 K^39^). At the lowest available temperature, *T* = 0.4 K, the ZFC diamagnetic signal value of 4πχ_*V*_(1-*N*) is close to −1 and provides evidence for full Meissner screening in LiPd_2_Si. A large difference between the FC and ZFC curves is frequently observed in polycrystalline superconducting samples.

The isothermal volume magnetization curves as a function of a magnetic field *M*_V_(*H*) measured for a range of temperatures (0.4 ≤ *T* ≤ 0.8 K) are displayed in Figure 4b. Assuming that the initial response to magnetic field is perfectly diamagnetic, the demagnetization factor *N* = 0.58 was found, consistent with the sample’s shape used in the magnetic measurements. The *M*_V_(*H*) curves initially show a linear dependence on the magnetic field and then drop to zero near the critical field. This is not a rapid drop, which is expected for type-I superconductors. This may be caused by the occurrence of intermediate state due to sample shape effect (demagnetization factor) as well as sample inhomogeneity. A similar feature has been observed for isostructural and isoelectronic LiPd_2_Ge and other type-I superconductors, such as KBi_2_,^60^ ScGa_3_,^61^ LuGa_3_,^61^ LiBi,^62^ and ReAl_6_.^63^

Low-temperature resistivity ρ(*T*) is shown in Figure 4c. A drop to zero is observed at the superconducting transition. The onset of the drop occurs at a temperature slightly higher than the one observed in the heat capacity and magnetization measurements. It is a typical behavior for a polycrystalline superconductor, as the drop of resistivity caused by the formation of a filamentary superconducting path usually occurs before the bulk of the sample becomes superconducting.

The residual resistivity ratio RRR = ρ(300 K)/ρ_0_ (where ρ_0_ is the residual resistivity just above the onset of superconducting transition; see Figure S2 of the Supporting Information) is 2.3, much lower than that measured for the LiPd_2_Ge sample (RRR = 14).^39^ Although the RRR of different materials is not directly comparable, such a high difference may suggest the presence of a stronger structural disorder in the measured LiPd_2_Si sample.

**Figure 5:**
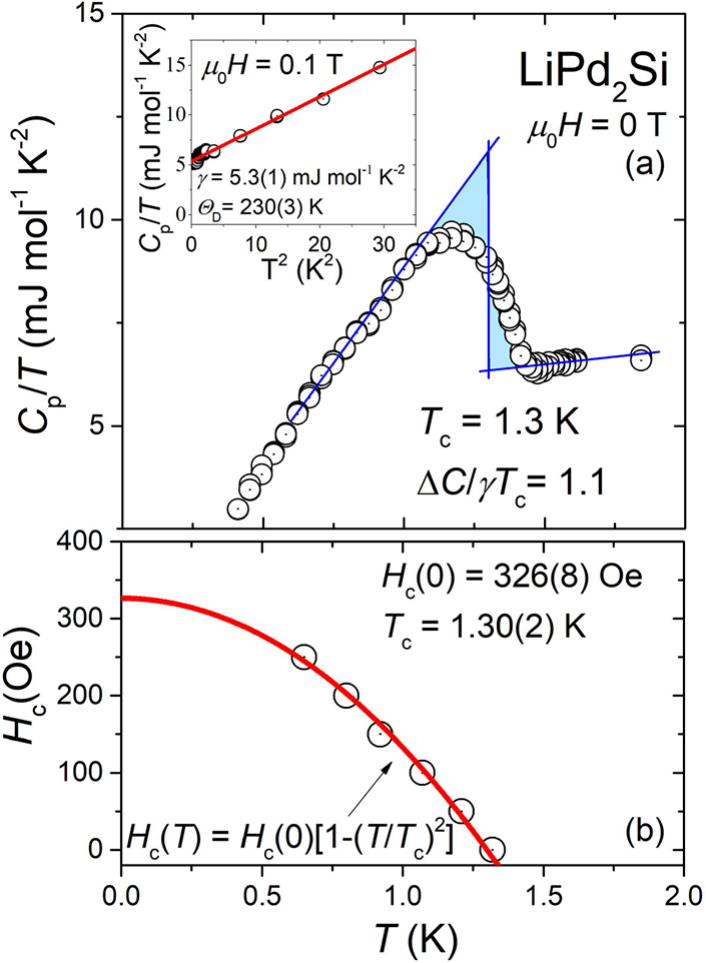
Panel (a) shows the temperature dependence of the specific heat in the vicinity of the superconducting transition. Equal entropy construction (blue lines) was used to estimate *T*_c_ = 1.3 K (in good agreement with magnetization results; see Figure 4). Inset: *C*_p_/*T* vs *T*^2^ measured in a 0.1 T applied magnetic field. The red solid line represents the linear fit used to estimate the values of the electronic and phonon specific heat coefficients. Panel (b) displays the critical field *H*_c_(*T*) determined from *C*_p_(*T*) (open circles).

To get important information about the superconducting transition, we carried out specific heat, *C*_p_(*T*), measurements at applied magnetic fields of both μ_0_*H* = 0 and 0.1 T. The inset of Figure 5a displays the temperature square dependence of *C*_p_/*T*, under an applied magnetic field of μ_0_*H* = 0.1 T. In the normal state, in which bulk superconductivity is completely suppressed, the experimental data can be fitted using the formula *C*_p_/*T* = γ + β*T*^2^, where γ is the Sommerfeld coefficient ascribed to the electronic contribution and β is the phonon specific heat coefficient. The fit is shown as a solid red line and gives γ = 5.3(1) mJ mol^–1^ K^–2^ and β = 0.32(1) mJ mol^–1^ K^–4^. The relatively low value of the Sommerfeld coefficient suggests weak electronic correlations. Then, the Debye temperature Θ_D_ was evaluated from β via a simple Debye model: , where *R* = 8.31 J mol^–1^K^–1^ and *n* = 2,^39^ yielding 230(3) K. The obtained values of γ and Θ_D_ are in good agreement with the previous report,^39^ in which data were collected only down to *T* = 1.8 K. The main panel of Figure 5a shows the low-temperature specific heat *C*_p_/*T*(*T*) of LiPd_2_Si in zero magnetic field. The presence of a well-defined jump in specific heat indicates bulk superconductivity with *T*_c_ = 1.3 K, which is consistent with the value obtained from the magnetic measurements. The normalized specific heat jump at the critical temperature, Δ*C*/γ*T*_c_, is 1.1, slightly lower than the value expected by BCS theory (1.43).

Having the estimated Debye temperature Θ_D_, the electron–phonon coupling constant λ_e-p_ can be estimated from the inverted McMillan formula:^64^
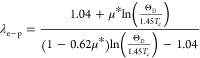
where μ* is the screened Coulomb pseudopotential parameter, typically taken as μ* = 0.13^19^ (in typical BCS superconductors, μ* varies within a narrow range of 0.10–0.14, as evidenced both by theoretical estimations and experimental measurements).^65,66^ Taking *T*_c_ = 1.3 K and Θ_D_ = 230 K, we obtain λ_e-p_ = 0.49, which is slightly lower than λ_e-p_ for an isoelectronic LiPd_2_Ge (λ_e-p_ = 0.56^39^). It is, however, worth noting that the estimation of λ_e-p_ depends on the assumed value of μ*. A relatively low value of the electron–phonon coupling constant as well as a small Δ*C*/γ*T*_c_ indicates that LiPd_2_Si is a weakly coupled BCS superconductor.

In addition, having the electron–phonon coupling constant and the Sommerfeld coefficient, the noninteracting density of states at the Fermi level DOS (*E*_F_) can be estimated from
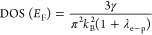
where k_B_ is the Boltzmann constant. The obtained DOS (*E*_F_) = 1.5 states/eV/f.u. is in a reasonable agreement with the value of DOS (*E*_F_) = 1.7 states/eV/f.u. from ab initio electronic structure calculations (see Figure 7).

Figure S3 in the Supporting Information shows the heat capacity measurements under applied magnetic fields. The magnitude of the discontinuity and the transition temperature decrease as the strength of the applied magnetic field is increased. The width of the transition also becomes broader in higher magnetic fields. The specific heat jumps (Δ*C*_p_/*T*_c_) measured under zero-field and 50 Oe (the lowest applied field) are comparable, suggesting crossover from the first- to second-order phase transition, expected in type-I superconductors.^60,62,67,68^

Figure 5b depicts the temperature dependence of critical field *H*_c_(*T*), with data points taken from the *C*_p_(*T*) measurements. The latest were used to the fit by a formula:
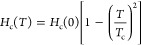
where *T*_c_ is the superconducting critical temperature, and *H*_*c*_(0) is the critical field at 0 K. The fit represented by a red solid line yields *T*_c_ = 1.30(2) K and a zero-temperature value of *H*_*c*_(0) = 326(8) Oe.

All of the extracted normal and superconducting state parameters are listed in Table 1.

**Table 1:** Comparison of the Physical Properties Reported for LiPd_2_Z (Z = Si, Ge, Sn)^a^

	LiPd_2_Si	LiPd_2_Ge^39^	LiPd_2_Sn^39^
*T*_c_ (K) exp./predicted	1.3/0.76^39^	1.96/1.5	-b/0.25
*H*_c_(0) (Oe)	326(8)	342	-
γ	5.3(1)	5.8(1)	4.4(1)
λ_e-p_^c^	0.49	0.56	-
Θ_D_ (K)	230(3)	194(3)	168(1)
DOS (*E*_F_)^d^	1.52	1.60	-
Δ*C*/γ*T*_c_	1.1	1.38	-

a Data for LiPd_2_Ge are taken from ref (39).

b “-” Superconductivity not observed down to *T* = 0.4 K.

c Estimated, assuming μ* = 0.13.

d Estimated from the experimental values of *T*_c_ and λ_e-p_.

**Figure 6:**
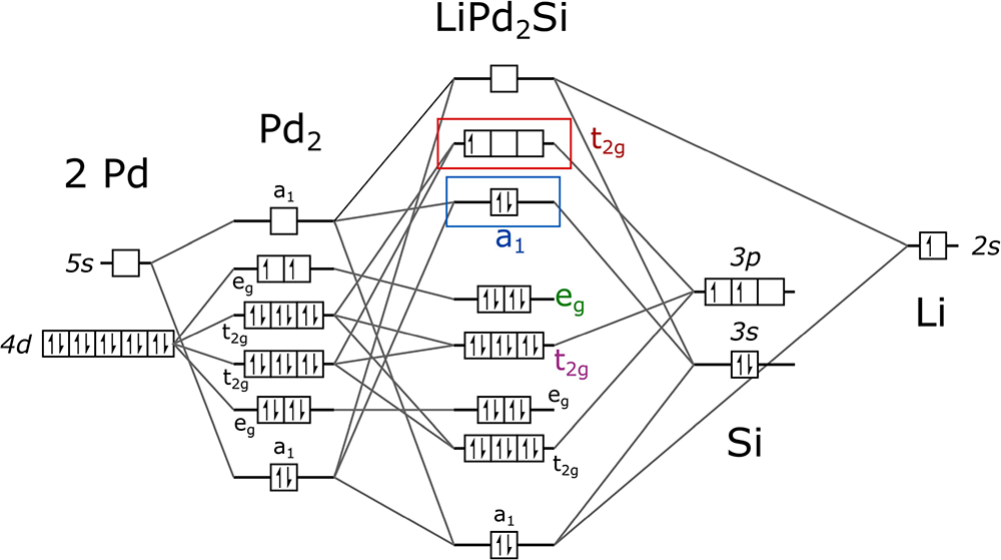
Crystal orbital scheme for LiPd_2_Si, presenting the electronic structure of the Heusler phase as an effect of interaction between the cubic Pd cage (left) and the Si and Li atoms. A nondegenerate CO (blue), derived from antibonding Pd–Si *s* interaction, is highlighted in blue. These orbitals form the first branch of the Fermi surface (see Figure 7e). The triply degenerate antibonding Pd *d–*Si *p* orbital (red) contributes to the remaining two FS branches. Orbital labels are colored consistently with Figure 7. Note that in the extended solid, the two highlighted orbitals (*a*_1_ and *t*_2g_) are empty at the Γ point (Figure 7), but the bands formed by them remain partially filled in other parts of the Brillouin zone.

The crystal orbital (CO) picture (Figure 6) of LiPd_2_Si can be described starting with a cubic Pd cage. The 4*d* and 5*s* atomic orbitals (AOs) of Pd interact, forming an electronic “backbone” which is for the most part preserved in the complete Heusler phase. Cage orbitals of the *e*_g_ symmetry do not strongly interact with Si *s/p* and Li *s* AOs and form rather narrow bands below *E*_F_. Si 3*p* interacts with Pd cage *t*_*2*g_ orbitals, changing the order of *t*_*2*g_ and *e*_g_ COs and forming a triply degenerate Pd *d*–Si *p* antibonding orbital (highlighted in red), which is populated by the single electron donated by Li.

**Figure 7:**
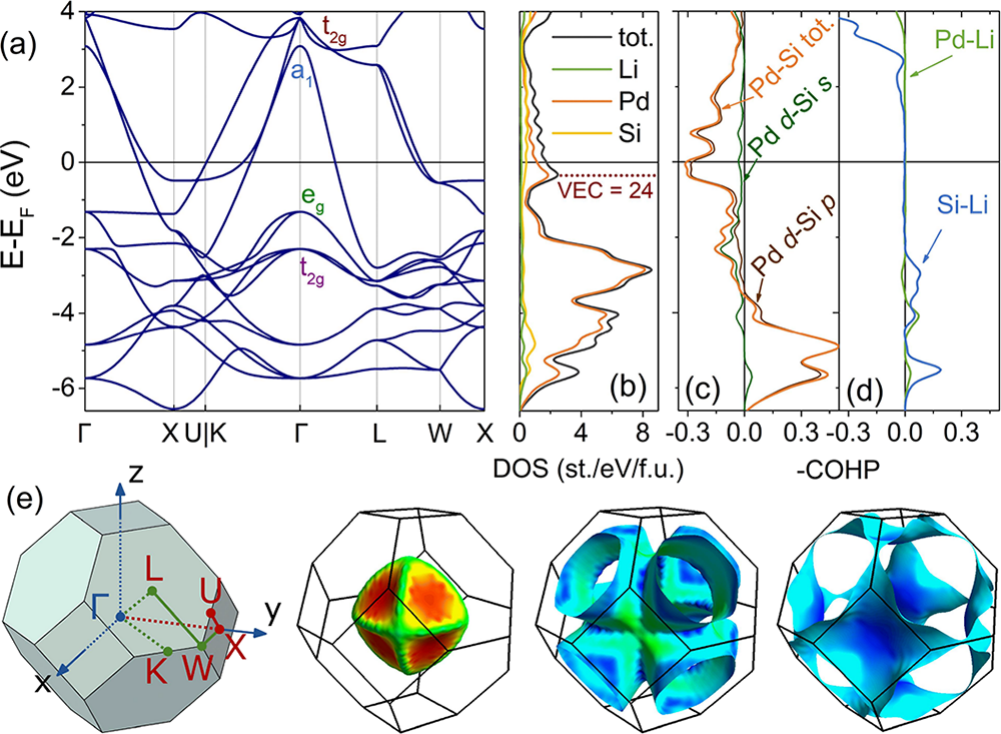
Results of DFT calculations of the electronic structure of LiPd_2_Si. Panel (a) shows the band structure with three individual bands (their symmetry at the Γ point is labeled in red and blue) crossing the Fermi level, forming three hole-like Fermi surface branches. Two weakly dispersive sets of bands (labeled green and purple) can be derived from the crystal orbitals of the Pd cage (Figure 6). Labeling of high-symmetry points is shown in panel (e). Panel (b) shows the electronic DOS. The highest of the three broad peaks between *E* = −6 and −2 eV below *E*_F_ is contributed almost exclusively by Pd *d* states, while the lower two show a stronger Si contribution, in accordance with CO considerations (Figure 6). The DOS (*E*_F_) = 1.7 states/eV/f.u. and is derived from the antibonding interaction of Pd and Si AOs, as shown in Panel (c). The negative value of −COHP for Pd *d* −Si *s/p* around *E*_F_ is consistent with the inferred antibonding character of the topmost occupied orbitals (Figure 6). Panel (d) shows the −COHP for Pd–Li and Si–Li pairs, suggesting that the antibonding Li-derived states lie well above *E*_F_, consistent with the polar nature of Li-(Pd_2_Si) bonding and highlighting the role of Li as an electron donor. Panel (e) shows the Brillouin zone (BZ) of a face-centered cubic lattice with high symmetry points shown in red (BZ center – Γ – in blue). Red and green lines are the paths through the BZ that were plotted in Panel (a). The three branches of FS are colored according to the increasing Fermi velocity from blue to red. The first branch derives from (Pd_2_) a_1_–Si *s*–Li s interaction, while the remaining two are from (Pd_2_) t_2_ -Si *p*.

This picture is consistent with the results of DFT calculations on LiPd_2_Si presented in Figure 7. The assumed order of COs agrees with the order of eigenvalues in the Brillouin zone (BZ) center Γ (Figure 7a). States ca. 2 eV below the Fermi level (*E*_F_) are almost completely contributed by Pd, while the DOS (*E*_F_) bears both strong Pd and Si contributions (Figure 7b). Li orbitals are mostly empty, with a peak of Li-derived DOS lying ca. 4 eV above *E*_F_. The crystal orbital Hamilton population analysis (−COHP, Figure 7c,d) shows that the interactions of Pd and Si contributing to the DOS (*E*_F_) are of an antibonding nature.

The topmost, partially filled *t*_*2*g_ CO forms the latter two of the three Fermi surface (FS) branches (see Figure 7a,e), while the first branch derives from a Pd–Si antibonding *a*_1_ orbital (blue) resulting from the interaction of Pd cage *a*_1_ orbital, Si 3*s* AO, and, to a lesser extent Li 2*s* AO. Li acts mostly as an electron donor to the CaF_2_-type Pd_2_Si sublattice, as evidenced by DOS and −COHP (Figure 7b,d). The calculated Bader charges are Li^+0.83^(Pd^–0.61^)_2_Si^+0.39^, suggesting that in addition to a strong electron transfer from Li to Pd, the Si atom also donates electron density to the Pd cage.

LiPd_2_Si is one of the two Heusler superconductors (SCs) containing 25 valence electrons (1 + 2 × 10 + 4) per formula unit.^24^ Most of the 34 Heusler SCs show valence electron count *VEC* = 26–29,^19,20,24,30,31^ all of them featuring the same electronic “backbone” of Ni-, Pd-, Pt-, or Au-based cubic cage interacting with an electropositive early transition metal (TM) or lanthanide (only 3 *s*-block metal-bearing SC Heusler compounds are reported) and a *p*-block metal/metalloid. In the remaining two superconducting phases, LiGa_2_Rh and LiGa_2_Ir^31,69^ (both with VEC = 16), the cubic cage is built from *p*-block atoms resulting in a qualitatively distinct electronic structure.

**Figure 8:**
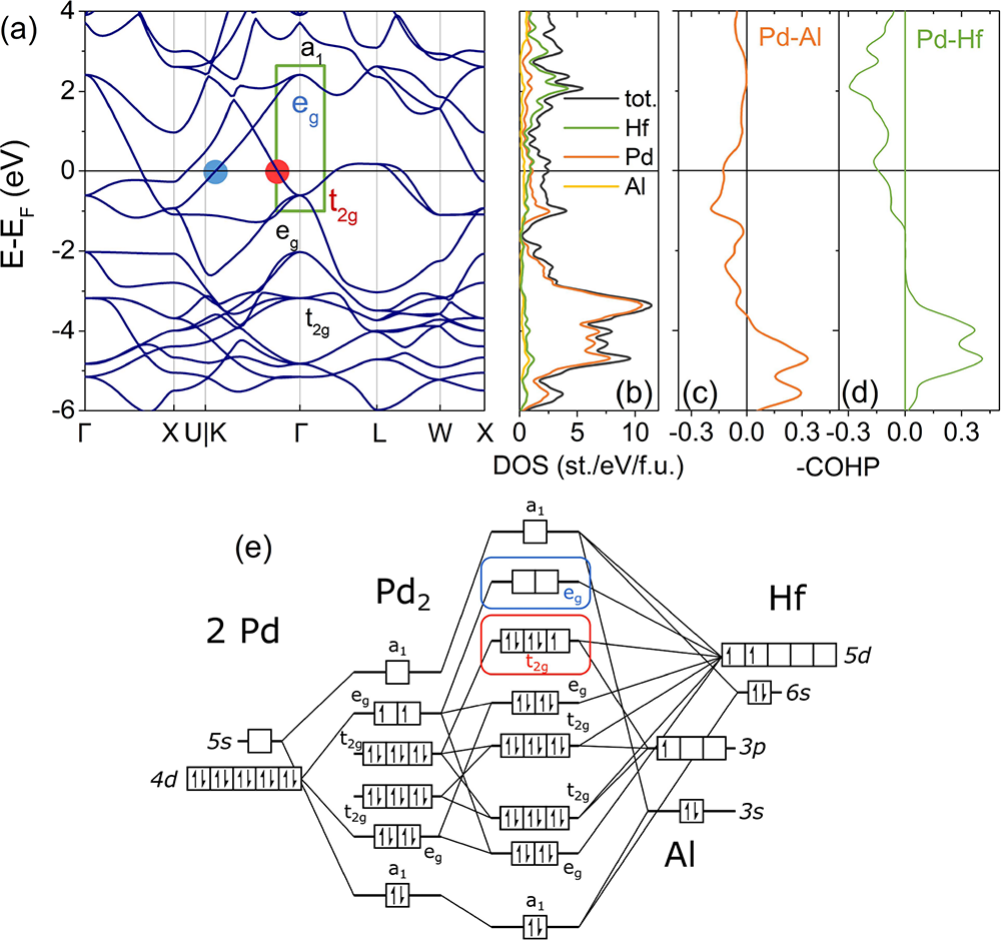
Panel (a) shows the calculated band structure of HfPd_2_Al. There are three bands crossing the Fermi level (highlighted with a green rectangle): one derived from the *e*_g_ crystal orbital (blue; see also panel (e)), forming a set of hole pockets, and two electron-like derived from *t*_2g_ CO (red). Colored circles mark the points along the K-Γ line where the respective bands cross the Fermi level. As in LiPd_2_Si (Figure 7a), the two sets of weakly dispersive bands positioned below the Fermi level (*E* – *E*_F_ from ca. −4 to −2 eV, labeled in black) can be derived from the Pd cage orbitals. The overall shape of the DOS (panel (b)) is similar to the LiPd_2_Si case; however, the DOS (*E*_F_) has a significant Hf contribution (while the Li contribution to DOS (*E*_F_) is negligible). COHP (c,d) shows that the bands crossing the Fermi level have Pd–Al and Pd–Hf antibonding characters. Panel (e) shows a crystal orbital scheme. Note that some of the high-energy (unoccupied) orbitals were omitted for clarity.

Besides the lower electron count, the major difference between the LiPd_2_Si and the VEC = 27 superconducting compounds results from the difference of frontier orbitals of Li (*s*) and TM/lanthanide (*s* and *d*). This results in a singly degenerate orbital, which contributes to the Fermi surface. On the contrary, in the majority of Heusler superconductors where a transition metal or lanthanide atom occupies the 4*a* site, the *a*_1_ orbital is much higher in energy, and its associated band does not cross the Fermi level (see Figure S4 of the Supporting Information for sample band structures of Heusler superconductors and Figure 8 for the band structure, DOS, COHP, and MO diagram of HfPd_2_Al).

The difference between the electronic structures of LiPd_2_Si and LiPd_2_Ge^39^ (VEC = 25) and the remaining Heusler SCs is not merely due to electron count but rather stems from different bonding situations, making the two compounds a special subgroup of the Heusler SC family. Thus, considering just the frontier orbitals involved and the VEC, it may be interesting to investigate the Mg(Ni,Pd,Pt)_2_(Al,Ga,In) and Li(Ni,Pt)_2_(Si,Ge,Sn) phases as possible superconductors, especially since some of them were independently highlighted as possible superconductors in a recent machine learning study.^37^ Moreover, there are several CaF_2_-type compounds reported with VEC values close to 25. We believe that they might be driven to superconductivity by filling the voids with Li or Mg atoms (owing to their relatively small size). A good example is the Rh_2_As phase^70^ (VEC = 23), which is reported to superconduct below *T*_c_ = 1.1 K.^71^ The OQMD database^72,73^ predicts that its Li-bearing Heusler counterpart LiRh_2_As (id:450688; VEC = 24) is just slightly above the convex hull of Li–Rh–As (0.001 eV/atom). This suggests that the Heusler phase may be synthesizable. Tuning the VEC by adding Li would help to understand the details of the VEC-*T*_c_ behavior of Heusler compounds. MgRh_2_As (id:451326; VEC = 25) would likely be difficult to stabilize (convex hull distance: 0.127 eV/atom).

Keeping in mind the expected semiconducting behavior of VEC = 24 full Heusler compounds,^4^ one may be tempted to see LiPd_2_Z (Z = Si, Ge, Sn) as a semiconductor/semimetal phase that is driven metallic and superconducting by increasing the electron count, a situation quite common among the known superconductor groups.^74,75^ However, our electronic structure calculations contradict this simple yet admittedly elegant picture in two ways: (1) a rigid band calculation with an electron count decreased to 24 for LiPd_2_Si shows no band gap at or around the Fermi level (Figure 7b), and (2) the order of electronic states/crystal orbitals is different in LiPd_2_Si and VFe_2_Al. As for (1), the calculated *E*_F_ for VEC = 24 would actually lie at a peak of DOS that results from a flat band along the U–X–W BZ path (Figure 7a,b), suggesting electronic instability. In the case of (2), the band gap appears in VFe_2_Al between the fully occupied *t*_2*g*_ triplet and *e*_g_ doublet.^4^ In LiPd_2_Si, there are two frontier COs: *a*_1_ and *t*_2g_, derived from the Pd cage *a*_*1*_ orbital interacting with Si *s* and Li *s* states, and two cage *t*_2g_ orbitals interacting with Si *p* states, respectively. Bands formed by both COs are partially filled, and a shift of the Fermi level that would result from a hypothetical removal of one electron per f.u. would only change the shape of the Fermi surface but not lead to a semiconducting behavior. The different order of COs derives from different frontier orbitals involved in the two compounds: V *d* states versus Li *s*. The importance of *d–d* interaction in the formation of band gap was extensively discussed in the literature for half Heusler phases.^76−78^ We recently showed that due to the lack of *d*–*d* hybridization, *s*-metal bearing half Heusler phases including MgPdSb, MgAgAs (and others), are robustly metallic even at the “closed shell” electron count (VEC = 18).^79^ Extension of the VEC–*T*_c_ relation to all known Heusler compounds is thus an oversimplification that neglects the differences in the bonding situation.

It is also worth noting that, while the “24-electron rule” helps to rationalize the semiconducting properties of VFe_2_Al,^4^ the number of reported *VEC* = 24 full Heusler compounds is limited to 5 (semiconducting VFe_2_Al, VFe_2_Ga^26,80,81^ and TiFe_2_Sn,^82^ semimetallic NbRu_2_Al,^83^ and half-metallic ferromagnet WMn_2_Sn^84,85^).

The Pd–Si states at the Fermi level are antibonding (Figure 7c). This supports the hypothesis that the occurrence of superconductivity is correlated with the occupation of antibonding electronic states,^74,86−93^ leading to a kind of “electronic strain” that can also result in a structural distortion^94^ or ferromagnetism.^95,96^ This is consistent with the general observation that in many cases superconductivity arises when a crystal structure distortion (such as charge density wave order) or magnetic transition is suppressed by tuning a system using chemical doping or pressure.^75,97−99^

## Conclusions

We have investigated the low-temperature physical properties of LiPd_2_Si, which belongs to the Heusler family (space group *Fm3̅m*, No. 225). According to ab initio calculations, LiPd_2_Si was expected to reveal superconducting behavior below *T* = 1 K.^39^ Specific heat and magnetic measurements performed down to low temperatures prove bulk superconductivity below *T*_c_ = 1.3 K, which is higher than that predicted. LiPd_2_Si is, therefore, the second superconductor in the Heusler family containing 25 valence electrons.

Detailed studies of the superconducting properties show that LiPd_2_Si is a weakly coupled BCS superconductor with an electron–phonon coupling constant λ_e-p_ = 0.49 and a heat capacity anomaly Δ*C*/γ*T*_c_ = 1.1. Moreover, as in the case of the isostructural LiPd_2_Ge, our results of the field dependence of isothermal magnetizations suggest a type-I superconductivity in LiPd_2_Si. We did not observe a superconducting transition in LiPd_2_Sn down to *T* = 0.4 K, in agreement with the earlier predictions.^39^

Almost all Heusler superconductors share the common structural motif of a Ni-, Pd-, Pt-, or Au-based cubic cage, with the overlapping *d* orbitals forming their electronic “backbone”. However, the LiPd_2_Z (Z = Si, Ge)^39^ and MgPd_2_Sb^24^ phases show distinctive features in the electronic structure when compared to the remaining Heusler superconductors. This is due to the fact that an *s*-block metal occupies the site that in other compounds is filled with an early transition metal or a rare-earth metal. While the rigid band approximation-based VEC–property relationship in Heusler compounds is a useful predictive tool,^4,19,24,100^ one has to include another important factor which is the type of interacting frontier orbitals, as some of the Heusler system features depend strongly on the presence of *d–d* interaction within the CaF_2_-type sublattice.

We postulate that VEC = 25 Heusler phases are interesting and largely unexplored candidates in the search for new superconducting intermetallic compounds.

## Supporting Information Available

XRD patterns of LiPd_2_Si, unit cell, and crystal structure parameters for LiPd_2_Si; low-temperature heat capacity under applied magnetic fields; and band structures of YPd_2_In, LuPt_2_In, ZrNi_2_Ga, and HfPd_2_Al Heusler compounds (PDF)

## Abbreviations

AO: atomic orbital
BCC: body-centered cubic structure
BCS: Bardeen–Cooper–Schriefer theory
BFGS: Broyden–Fletcher–Goldfarb–Shanno algorithm
CO: crystal orbital
DFT: density functional theory
DOS: density of (electronic) states
FC: field cooled
FCC: face-centered cubic structure
FS: Fermi surface
MO: molecular orbital
PBE GGA: Perdew–Burke–Ernzerhof generalized gradient approximation
SC: superconductor
VEC: valence electron count
XRD: X-ray diffraction
ZFC: zero-field cooled
