# Supplementary material for: Superconductivity–Electron Count Relationship in Heusler Phases—the Case of LiPd2Si
Source: Chem Mater. 2024 Feb 15;36(4):1870–9. doi: 10.1021/acs.chemmater.3c02398 (PMC10902818; doi:10.1021/acs.chemmater.3c02398)
Supplement: Supplementary file 1 — cm3c02398_si_001.pdf [file cm3c02398_si_001.pdf]

Superconductivity-Electron Count Relationship in Heusler Phases - the Case of $LiPd_2Si$

Karolina Górnicka[1], Xin Gui[2], Juan R. Chamorro[3], Tyrel M. McQueen[4], Robert J. Cava[2], Tomasz Klimczuk[1,$] and Michał J. Winiarski[1,*]

[1] Faculty of Applied Physics and Mathematics and Advanced Materials Centre, Gdansk University of Technology, ul. Narutowicza 11/12, 80-233 Gdańsk, Poland,

[2] Department of Chemistry, Princeton University, Princeton, NJ 08540, USA

[3] Materials Department and Materials Research Laboratory, University of California, Santa Barbara, Santa Barbara, CA 93106, USA

[4] Department of Chemistry, Department of Physics and Astronomy, Department of Materials Science and Engineering, and Institute for Quantum Matter, Johns Hopkins University, Baltimore, MD 21218, USA

$ tomasz.klimczuk@pg.edu.pl

* michal.winiarski@pg.edu.pl

Supporting Information

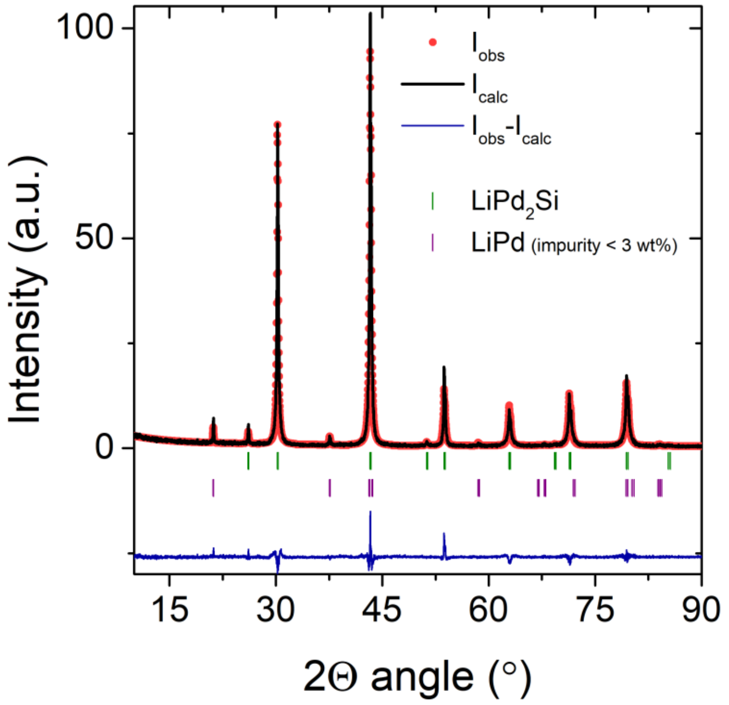

Fig. S1 Rietveld fit (black) to the powder x-ray diffraction pattern of $LiPd_2Si$ (red points). Green ticks mark the expected position of Bragg peaks for the main phase, purple – for the LiPd impurity (2.9(1)wt% based on the fit).

Tab. S1 Unit cell and structural parameters of $LiPd_2Si$ obtained by Rietveld fit presented in Fig. S1. *R*-factors given in the table are conventional Rietveld (background corrected) *R*-factors calculated only for points with Bragg contributions [1].

$LiPd_2Si$<br>room temperature powder x-ray diffraction (Cu K_α)				
Unit cell parameter (Å)	5.9052(2)			
Unit cell volume (Å^3)				
Formula weight	247.87			
Z	4			
Calculated density	8.00			
Fit reliability factors:				
R_p (%)<br>R_{wp} (%)<br>R_{exp} (%)<br>χ^2	13.8<br>16.5<br>12.35<br>1.79			
Atom	x	y	z	B_{iso} (Å^2)
Li	0	0	0.5	1.3(9)
Pd	0.25	0.25	0.25	0.78(4)
Si	0	0	0	0.5(1)

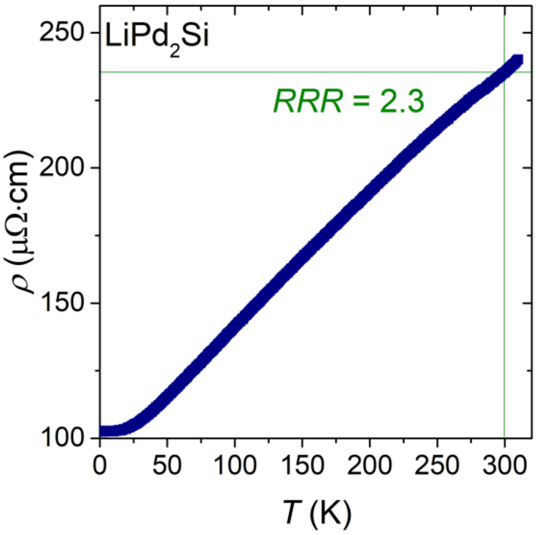

Fig. S2 Resistivity of LiPd$_2$Si in a temperature range T = 2 K to 310 K. The residual resistivity ratio ($\rho_{300\ K}$ / ρ_0 = 2.3).

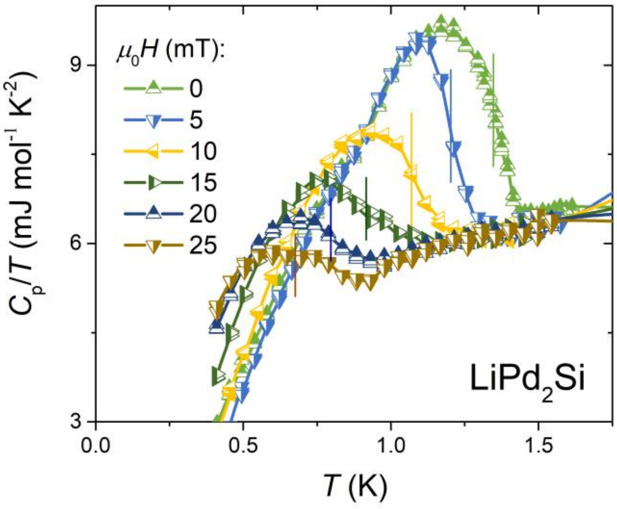

Fig. S3 The dependence of the specific heat C_p on temperature in applied magnetic fields H up to 250 Oe.

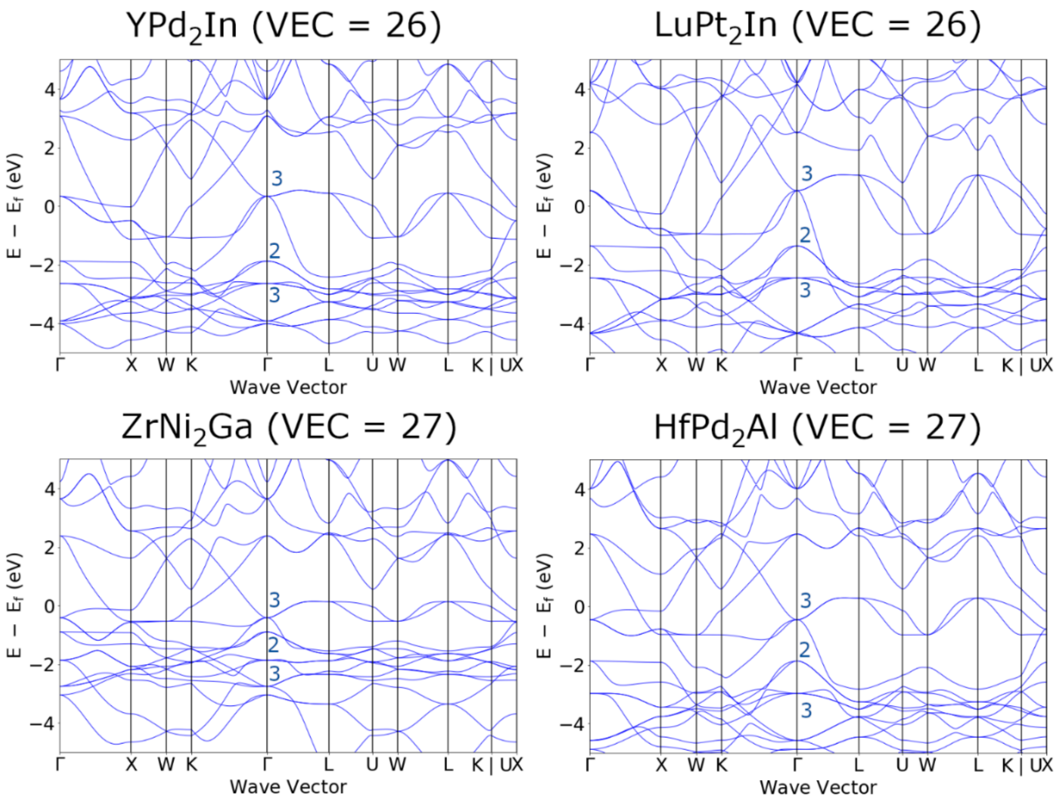

Fig. S4 Band structures of YPd_2In, $LuPt_2In$, $ZrNi_2Ga$, and $HfPd_2Al$ Heusler compounds taken from the Materials Project database [2] (ID numbers: mp-568880, mp-1185489, mp-22580, mp-16501, respectively). Numbers in blue show the degeneracy of bands around the E_F at the Brillouin zone center (Γ point). No singly-degenerate band is found in the vicinity of the E_F in contrast with alkali/alkaline-earth bearing $LiPd_2Si$ (Fig. 5), $LiPd_2Ge$ (see ref. [3]), and $MgPd_2Sb$ (see ref. [4]) Heusler.

References:

(1) Huot, J.; Černý, R. Neutron Powder Diffraction. In *Neutron Scattering and Other Nuclear Techniques for Hydrogen in Materials*; Fritzsche, H., Huot, J., Fruchart, D., Eds.; Neutron Scattering Applications and Techniques; Springer International Publishing, 2016; pp 31–89. https://doi.org/10.1007/978-3-319-22792-4_3.

(2) Jain, A.; Ong, S. P.; Hautier, G.; Chen, W.; Richards, W. D.; Dacek, S.; Cholia, S.; Gunter, D.; Skinner, D.; Ceder, G.; Persson, K. A. Commentary: The Materials Project: A Materials Genome Approach to Accelerating Materials Innovation. *APL Materials* **2013**, *1* (1), 011002. https://doi.org/10.1063/1.4812323.

(3) Górnicka, K.; Kuderowicz, G.; Carnicom, E. M.; Kutorasiński, K.; Wiendlocha, B.; Cava, R. J.; Klimczuk, T. Soft-Mode Enhanced Type-I Superconductivity in $LiPd_2Ge$. *Phys. Rev. B* **2020**, *102* (2), 024507. https://doi.org/10.1103/PhysRevB.102.024507.

(4) Winiarski, M. J.; Kuderowicz, G.; Górnicka, K.; Litzbarski, L. S.; Stolecka, K.; Wiendlocha, B.; Cava, R. J.; Klimczuk, T. $MgPd_2Sb$: A Mg-Based Heusler-Type Superconductor. *Phys. Rev. B* **2021**, *103* (21), 214501. https://doi.org/10.1103/PhysRevB.103.214501.
